# NMR Magnetometer Based on Dynamic Nuclear-Polarization for Low-Strength Magnetic Field Measurement

**DOI:** 10.3390/s23104663

**Published:** 2023-05-11

**Authors:** Taoning Guo, Wei He, Cai Wan, Yuxiang Zhang, Zheng Xu

**Affiliations:** School of Electrical Engineering, Chongqing University, Chongqing 400044, China

**Keywords:** NMR, pre-polarization, low magnetic field measurement

## Abstract

Nuclear magnetic resonance (NMR) magnetometers are considered due to their ability to map magnetic fields with high precision and calibrate other magnetic field measurement devices. However, the low signal-to-noise ratio of low-strength magnetic fields limits the precision when measuring magnetic fields below 40 mT. Therefore, we developed a new NMR magnetometer that combines the dynamic nuclear polarization (DNP) technique with pulsed NMR. The dynamic pre-polarization technique enhances the SNR under a low magnetic field. Pulsed NMR was used in conjunction with DNP to improve measurement accuracy and speed. The efficacy of this approach was validated through simulation and analysis of the measurement process. Next, a complete set of equipment was constructed, and we successfully measured magnetic fields of 30 mT and 8 mT with an accuracy of only 0.5 Hz (11 nT) at 30 mT (0.4 ppm) and 1 Hz (22 nT) at 8mT (3 ppm).

## 1. Introduction

NMR (nuclear magnetic resonance) is considered the gold standard for high-precision measurements of high-strength magnetic fields [1,2,3,4,5,6]. NMR magnetometers are often used to measure magnetic field values, map spatial magnetic field distributions (for example, in the main magnet of the MRI equipment to measure the homogeneity and temperature drift of the main magnetic field) [7,8,9,10,11,12], and calibrate magnetic field measurement equipment based on other physical principles [13,14]. However, magnetometers have been limited by the low signal-to-noise ratio (SNR) in low-strength magnetic field conditions, resulting in poor performance in low-strength magnetic field applications.

The development of this magnetic resonance magnetometer holds great significance for both the study and industry fields. For instance, portable and mobile nuclear magnetic resonance imaging equipment [15,16,17,18,19,20] is a relatively new field that many schools and companies are constantly researching and exploring. The magnetic field of these devices is typically generated by a permanent magnet, and in order to make these devices more portable, the magnet size must be reduced, but this change is accompanied by a decrease in magnetic field strength. However, the limitations of magnetic field measurement tools have restricted the development of mobile nuclear magnetic imaging to lower field strengths. Mobile magnetic resonance imaging requires high uniformity of the magnetic field, and the precision of commonly used magnetic field measurement equipment is insufficient.

The only commercially available magnetic resonance magnetometer that can handle this task is the PT2026 produced by Metrolab; however, the minimum magnetic field measurement range of the PT2026 is only 38 mT. The serial magnetic resonance magnetometer designed in this paper fills the gap in high-precision low-field magnetic field measurement. It holds significant importance for the development of nuclear magnetic resonance imaging.

In the process of NMR magnetometer measurement, the magnetic field value is obtained by measuring the frequency. According to the Larmor formula, *ƒ*_0_ = (*γ*/2π) *B*_0_, for ^1^H nuclei, (*γ*/2π) = 42.57747892 MHz/T, the magnetic field value exhibits a linear correlation with the frequency, wherein the constant of proportionality is the gyromagnetic ratio (γ). Thus, the magnetic field value strictly depends on the frequency and is unaffected by factors such as temperature [21].

There are two categories of NMR magnetometers: the pulsed NMR type [22,23] and the continuous-wave NMR type. The pulsed NMR uses a broadband pulse to excite the sample, collects the sample’s free induction decay (FID) signal, performs Fast Fourier Transform (FFT) on the FID signal to obtain a frequency spectrum, and the peak of the spectrum is considered to be the frequency of the magnetic field. Pulsed NMR has high measurement accuracy and fast measurement speed, but the disadvantage with pulsed NMR is that it requires customization of the probe for different applications and also that the working frequency band of the probe is narrow. Continuous-wave NMR magnetometers typically seek and track the signal in a sample by sweeping the frequency or field. When the frequency or field of the sweep matches the nuclear resonance frequency in the sample, we can detect an NMR signal, thereby determining the magnitude of the magnetic field. Unlike pulsed NMR, continuous-wave NMR does not require customized probes for different magnetic fields, but its measurement accuracy and speed are far inferior. Both methods are limited by low SNR in low field strength and cannot always be used in such scenarios. To improve the measurement capability of NMR magnetometers for low fields, researchers have adopted methods such as large samples [14,24], optical or microwave hyperpolarization [25,26], and polarization [27,28,29,30].

In previous studies on dynamic nuclear polarization, the continuous wave method has been the most frequently used to excite the water samples, and the change in the magnetization vector of the liquid during the transfer process was detected to measure the strength of the magnetic field. Due to the complex effect of the radiofrequency perturbation on the magnetization vector, magnetic field measurement is also susceptible to environmental factors, which has resulted in a relatively slow development in this field.

In 1978, Pendlebury [29] improved the previously existing [31] NMR magnetometer technology by building a double-coil flow-through NMR magnetometer using the averaging concept. This method involves placing two perturbation coils in the magnetic field to be measured, and the detected magnetization vector change is the superposition effect of two resonance effects, which results in an improvement of the detection sensitivity. By placing a perpendicular excitation coil before and after the magnetic field to be measured and applying sinusoidal oscillation, when the frequency of the RF excitation satisfies the resonance condition of the magnetic field to be measured, the hydrogen protons in the water absorb RF energy, causing a change in the macroscopic magnetization vector, which can be obtained by the self-difference receiver. This method can simultaneously obtain information on the central field strength and the homogeneity of the magnetic field distribution in the area to be measured, but the spatial measurement resolution is relatively low. In addition, the double-coil structure will reduce the accuracy of the measurement results, and there can be a problem with side peaks in the frequency response. In 1996, Woo [28] developed a flow-type nuclear NMR magnetometer using a single perturbation coil, two magnets, and a commercial self-differencing receiver. He simplified and systematically explained the mathematical and physical principles involved in the measurement process and proposed a sensitivity optimization method based on determining the optimal perturbation strength by detecting the NMR signal intensity at a frequency offset of 10 Hz from the center frequency. This method improved the SNR and facilitated detailed analysis of the effect of excitation strength on the full-width at half maximum and signal amplitude of the measurement results. In 2015, Davydov [32,33] et al. improved the commercial self-compensated magnetometer by increasing the signal-to-noise ratio in the detection of magnetization vectors through signal superposition. They extended the pipeline length to 69m using a liquid sample and a fast cycling system and achieved rapid measurement of magnetic field distribution and variation at different positions with a single system. By installing multiple radio frequency excitation coils during the polarization and detection process and using electronic switching to apply excitation signals separately, the accuracy of rapid measurements was improved. However, due to the limited signal-to-noise ratio in signal detection, the margin of error was about 0.3%. However, the addition of toxic strong acid components to the flowing medium caused many problems. In 2018, Michal Ulvr [30] and others improved the measurement range of the flow-through NMR magnetometer (precession method), increased the measurement signal-to-noise ratio, and obtained new resonance frequency values analyzing the double-peak spectrum. This broadened the lower limit of NMR measurement with expanded uncertainty of 20 to 60 ppm. However, limited by the continuous wave frequency scanning excitation method, the measurement speed was slow, and an average measurement took about 30 min.

These methods have, to some extent, expanded the lower limit of NMR magnetometer measurement. As shown in Table 1, although the magnetic field strength measured in our experiment is not the lowest, our method has the highest precision and the fastest measurement speed. These advantages are attributed to the pulse wave method and are not present in other magnetometers that use continuous wave methods. Additionally, the size of the probe coil area is relatively small, which improves the spatial resolution of the measurement.

To address these concerns, we propose a new NMR magnetometer structure. Our main contributions are as follows:

- Proposed a method that combines pulsed NMR with dynamic nuclear polarization (DNP), which extends the measurement lower limit, improves measurement speed, and ensures measurement accuracy;
- Proposed a new magnetometer structure and simulated the measurement process, analyzing the influence of different factors on the signal-to-noise ratio;
- Constructed a complete measurement system and verified its accuracy, as well as improved the SNR of the magnetometer in low magnetic field measurements through experiments at different magnetic field strengths.

## 2. Simulation Computation and Methods

### 2.1. NMR Magnetometer Structure

**Figure 1:**
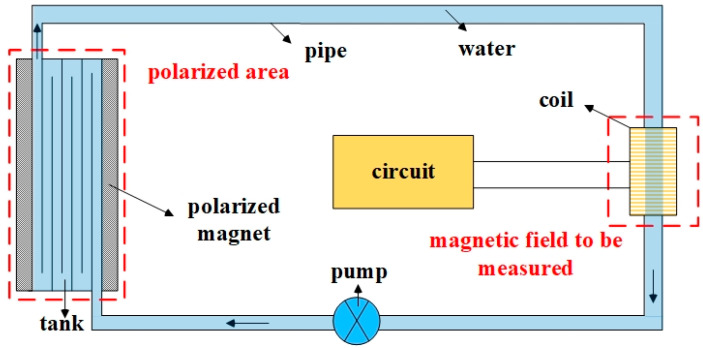
NMR magnetometer structure diagram.

As shown in Figure 1, the structural diagram of the flowing-water NMR magnetometer includes an NMR probe, a pre-polarization unit, a water sample transfer unit, and a control circuit. The probe operates within the magnetic field area to be tested and is responsible for exciting the water sample and the magnetic field to generate resonance and acquire the NMR signal. The probe includes a detection coil and a matching circuit. The pre-polarization unit operates in the water sample polarization area, which is responsible for pre-polarizing the water sample to increase a higher longitudinal magnetization vector of the sample. The pre-polarization unit mainly comprises a polarization magnet with a high magnetic field. The water transfer unit functions to form a closed water circuit so that the water sample can be quickly transferred from the polarization area to the detection area. The water sample transfer unit comprises a water pipe, a water pump, and a water tank arranged in the magnetic field area inside the polarization magnet. The control circuit is responsible for RF excitation and NMR signal reception and includes a spectrometer circuit and some analog circuits.

The flowing-water magnetometer is as follows: during the measurement, water flows through the polarization field and to the detection field, and the NMR signal passes through three stages: polarization, excitation, and detection [34,35].

1. Polarization

The macroscopic magnetization vector is the foundation of NMR, and the process of establishing the macroscopic magnetization vector in the sample by applying an external static magnetic field to the hydrogen protons is called polarization. In the flow-type NMR magnetometer, the polarization magnet produces a strong static polarization magnetic field *B_p_* to induce polarization of the hydrogen protons.

In the plane perpendicular to *B_p_*, there is no transverse magnetization vector due to the inconsistent proton precession phases. However, in the direction parallel to *B_p_*, a macroscopic magnetization vector *M*_0_ is established:

(1) $$M_{0}=\frac{\rho \gamma^{2}h^{2}}{4kT}B_{p}$$

Naturally, the larger the *B_p_* value, the more evident the level splitting, and the greater the intensity of the *M_p_*. The process of the macroscopic magnetization vector (reaching Boltzmann equilibrium) is not instantaneous and depends on the longitudinal relaxation time *T*_1_ of the sample:

(2) $$M_{p}=M_{0}(1-e^{-t_{p}/T_{1}})$$

where *M_p_* represents the macroscopic magnetization vector intensity of a sample when it flows out of the polarizing field, and *t_p_* represents the time it takes for the sample to flow through the polarizing magnetic field. *M*_0_ is the maximum longitudinal magnetization vector achieved with a polarizing magnetic field *B_p_*. During the *t_pd_* time, it takes to flow out of the polarizing magnetic field into the magnetic field to be measured. If the sample is not affected by interference, its macroscopic magnetization vector decays according to the *T*_1_ law:

(3) $$M_{d}=M_{0}(1-e^{-t_{p}/T_{1}})e^{-t_{pd}/T_{1}}$$

2. Excitation

The measurement field is configured with a time-division multiplexing coil, which, under the action of the control circuit, generates an RF excitation magnetic field *B*_1_ perpendicular to the magnetic field *B_m_* being measured. Protons at a low energy level absorb RF energy and undergo energy level transitions. When an RF magnetic field *B*_1_ is applied to the plane perpendicular to the measured magnetic field *B_m_* at the same Larmor frequency, the longitudinal magnetization vector *M_p_* flips around the B1 axis perpendicular to the plane. During the measurement process, a π/2 RF pulse to flip *M_p_* by 90 degrees is applied.

3. Detection

After the application of RF excitation, the transverse magnetization vector *M_xy_* undergoes a loss of phase due to the spin–spin interaction of the atomic nuclei, resulting in the composite vector gradually decaying to zero, according to the formula:

(4) $$M_{xy(t)}=M_{xy\max}e^{-t/T_{2}}$$

At this point, a free induction decay (FID) signal is induced at both ends of the coil, and the induced voltage signal satisfies the following formula:

(5) $$u\propto M_{0}\sin(\omega t)e^{-t/T_{2}{}^{*}}$$

where *T*_2_ is the transverse relaxation time, which represents the time it takes for a magnetization vector generated in the transverse plane to decay to zero. *T*_2_* is shorter than *T*_2_ because, in the measurement processes, there are factors such as field gradients and others that render the field inhomogeneous, causing the transverse magnetization to decay faster.

By collecting the voltage signals at both ends of the coil through a data acquisition circuit and performing FFT analysis, the frequency peak value obtained is the frequency of the measured field [36]. The magnetic induction intensity converted through the Larmor formula is the strength of the measured magnetic field.

(6) $$f_{0}=(\gamma /2\pi )B_{0}$$

For ^1^H nuclei, (*γ*/2π) = 42.57747892 MHz/T. The magnetic field value has a linear relationship with the frequency, and the proportionality coefficient is the gyromagnetic ratio *γ*, which is a constant.

### 2.2. Factors Affecting NMR

There are several influencing factors in the previously described process. First, there is the strength of the polarizing magnet. The stronger the polarizing magnetic field, the stronger the residual magnetization vector of the water sample flowing into the region to be measured and, therefore, the stronger the induced signal. The second factor is the length of the water pipe from the polarizing field to the detection field. With a constant flow rate, the shorter the water pipe, the smaller the loss of the magnetization vector during transfer. The third factor is the length of the probe coil. A longer coil can accommodate more NMR signals that correspond to the water sample, which can enhance the signal intensity. However, the drawback is that it leads to a decrease in the spatial resolution of the magnetic field and a decrease in the ability to measure inhomogeneous fields. The fourth factor is the transfer speed of the water sample, which is influenced by the flow rate of the water pump. When the flow rate of the water sample is too slow, the initial longitudinal magnetization vector of the pre-polarized sample, as well as its decay, approaches zero by the time it reaches the detection area, rendering the pre-polarization ineffective. On the other hand, when the flow rate of the water sample is sufficiently high, the excited water sample may not have enough time to acquire the NMR signal before being flushed out of the detection coil area. Therefore, there exists an optimal flow rate that maximizes the detected signal.

We conducted simulation calculations based on the established model under the following conditions: probe coil length *L_m_* = 3 mm, diameter = 5 mm, water pipe length from polarizing magnet to detection coil (*L_pd_*) = 2 m, polarizing magnetic field strength = 1.3 T, measurement field magnetic field strength = 30 mT.

We define the sample flow rate as *V_f_* (unit: mL/min), the length of the measurement coil covering the pipeline as *L_m_* (unit: m), and the length of the pipeline between the polarizing field and the detection field as *L_pd_* (unit: m).

(7) $$D=(1-t_{r}\frac{V_{f}{}_{}}{\pi r^{2}\times L_{m}})$$

(8) $$S_{}=\frac{M_{d}\times D}{M_{measure}}=\frac{M_{o}(1-e^{-t_{p}/T_{1}})e^{-t_{pd}/T_{1}}\times D}{\frac{\rho \gamma^{2}h^{2}}{4kT}B_{m}}$$

Assuming that the magnetic field being measured exists only in the probe coil region, the parameter *S* in the equation represents the ratio of the magnetization vectors corresponding to the polarized and non-polarized water samples, respectively, when the coil is detected. The letter *D* represents the residual water sample ratio in the detection area of the probe coil. When stimulating the flowing water sample, a portion of the stimulated sample is flushed out of the detection area, while the remaining portion is retained in the coil detection area and becomes the main source of the signal. Considering that in practice, the duration of the radio frequency pulse (*t_r_*) is about 5 μs, the equation for *D* is Formula (7).

**Figure 2:**
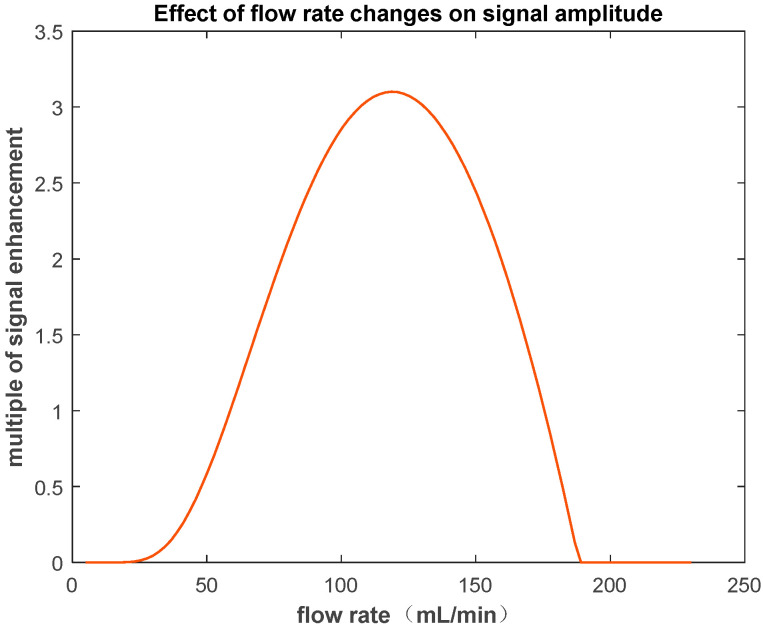
Relationship between NMR signal amplitude and flow velocity.

As shown in Figure 2, by calculating the enhancement factor of the NMR signal based on different flow rates, the optimal flow rate can be obtained.

**Figure 3:**
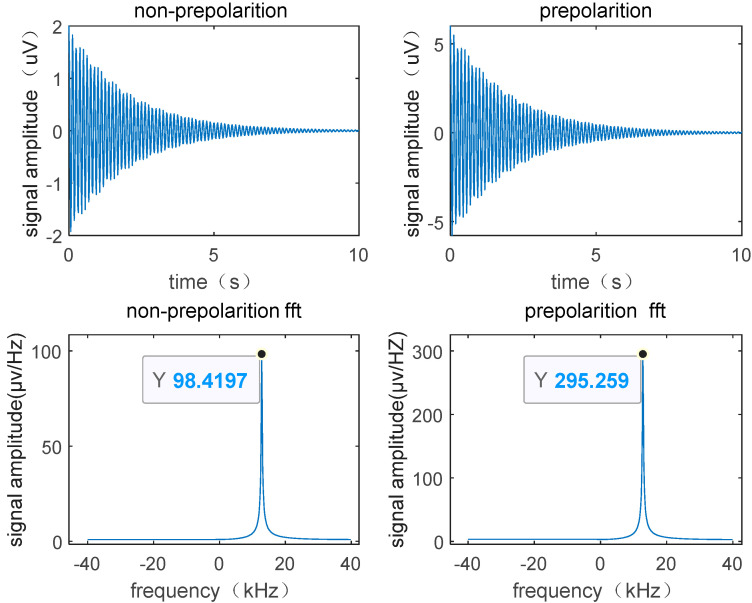
The effect of pre-polarization on the FID signal and the corresponding amplitude spectrum.

Simulating and analyzing the signals of polarized and non-polarized water samples at the optimal flow rate, as shown in Figure 3, it can be observed that with the same magnetic field strength, the peak value of the FID signal differs by a threefold difference between the peak values of the two. The spectrum of the pre-polarized water NMR signal peak is steeper compared to the un-polarized, as measured by the height-to-width ratio (H/W). H/W (un-polarized) = 31.6, H/W (polarized) = 94.8.

**Figure 4:**
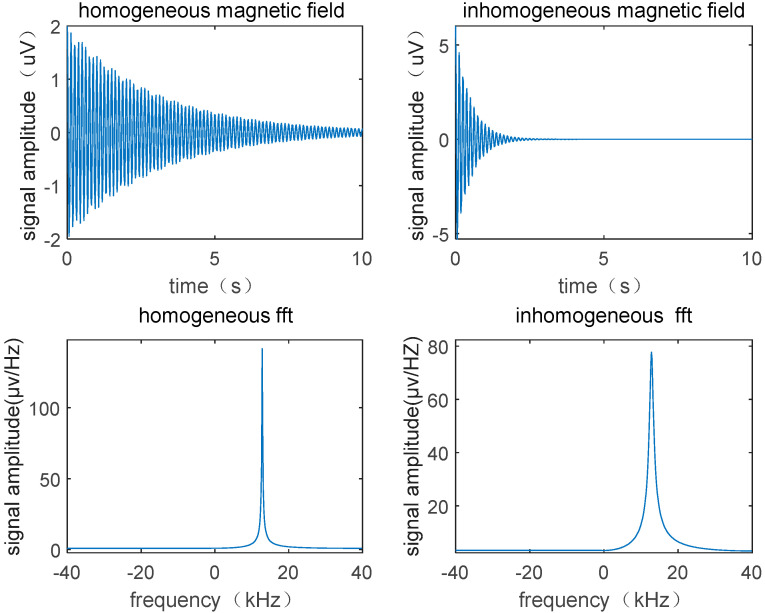
Influence of inhomogeneous magnetic field on signal.

The homogeneity of the magnetic field being tested has a significant impact on the magnetic field measurements. As shown in Figure 4, when the homogeneity of the magnetic field being test is poor, the FID signal decays faster, and the decay of the FID signal is primarily influenced by the time *T*_2_* (which is closely related to the inhomogeneous of the magnetic field). As the decay rate accelerates, the spectral lines in the frequency spectrum analysis become smoother, and the peak-to-width ratio of the FFT frequency spectrum analysis curve becomes smaller, which is counterproductive to our goal of sharpening the spectral lines and indicates that the measurement of the NMR magnetometer requires homogeneity restriction of the magnetic field. By reducing the size of the probe, the measurement ability of the flow-type magnetic resonance magnetometer can be improved for inhomogeneous fields. The smaller the size of the probe coil, the smaller the area of the magnetic field it excites, and the smaller the corresponding variation in magnetic field intensity within the area, which can significantly improve the *T*_2_* duration of the FID signal. In addition, reducing the size of the probe also has the advantage of improving spatial resolution.

## 3. System Design

The design of the continuous-flow NMR magnetometer involves three main parts: the magnetic circuit, the water circuit, and the electrical circuit.

**Figure 5:**
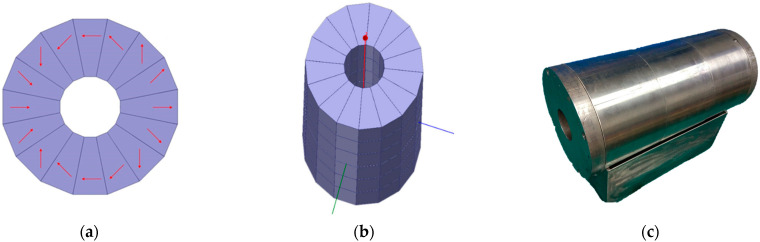
Halbach magnet structure and real picture (picture (**a**) shows the distribution of 16 magnets on one layer; picture (**b**) shows the structure of 6 layers; picture (**c**) shows the entity of the magnets).

In order to achieve low-strength magnetic field measurements, it is necessary to first pre-polarize the water sample to increase the size of the macroscopic magnetization vector. As shown in Figure 5, a ring-shaped Halbach magnet [37] composed of 16 blocks × 6 layers of trapezoidal neodymium iron boron magnets with different magnetization directions is used. The magnet structure is shown in Figure 5a,b, and the magnet itself is shown in Figure 5c. The outer diameter, inner diameter, height, and internal magnetic field strength of magnets are 17 cm, 5 cm, 30 cm, and 1.31 T, respectively.

**Figure 6:**
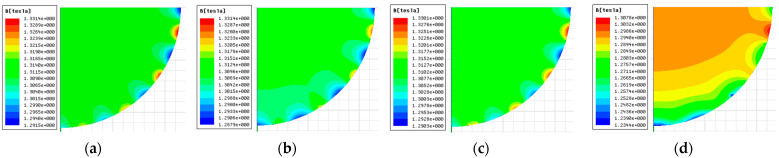
Simulation diagram of the magnetic field at different positions of the magnet (assuming that the height of the middle of the magnet axis is 0; (**a**) shows the 1/4 cross-sectional simulation of the interface with a height of 0; (**b**) shows the magnetic field at a height of 50 mm; (**c**) shows the magnetic field at a height of 100 mm; (**d**) shows the magnetic field at a height of 150 mm).

Due to a special arrangement of permanent magnets, the Halbach [38] magnet makes full use of the mutual constraint of magnetic field lines, allowing the entire magnet to be operated without the need for yokes. Moreover, the magnet has no external magnetic field due to its non-magnetic outer structure. The simulation results of the internal field strength of the magnet are shown in Figure 6a–d.

It can be seen that the central strength of the magnetic field is about 1.3 T. The closer to the central area, the more inhomogeneous the magnetic field distribution. The inhomogeneity mainly comes from the part of the cavity close to the inner wall magnet, and the closer to the outside of the cavity, the worse the inhomogeneity of the magnetic field distribution becomes. In the edge area of the cavity above 100 mm, the magnetic field strength changes significantly; its homogeneity distribution also becomes worse.

The waterway design includes a water tank arranged inside the polarizing magnet, with S-shaped water troughs inside the tank; the NMR probe is made of glass capillary tubes and a variable-speed water pump. These parts are connected in series through water pipes to form a complete water circuit

**Figure 7:**
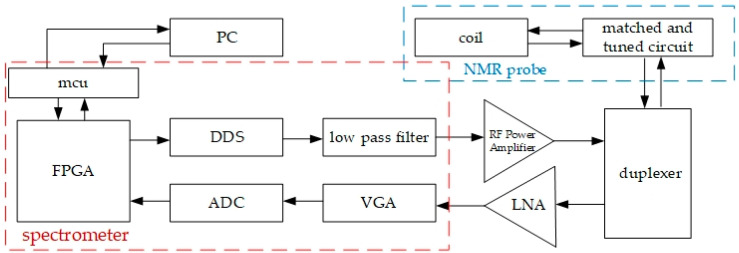
Circuit diagram of NMR magnetometer.

Figure 7 is the overall circuit structure diagram of the circuit section. When the magnetic sensing probe reaches the magnetic field to be measured, the spectrometer [39,40] outputs a low-power RF signal corresponding to the Larmor frequency of the hydrogen atomic nucleus that is proportional to the strength of the magnetic field to be measured. After amplification by an RF power amplifier, the duplexers isolate the high-power RF signal and directly transmit it to the tuning and matching network of the probe, generating an RF field perpendicular to the magnetic field direction of the coil and causing the macroscopic magnetic angle deviation of the nucleus to demonstrate a nuclear magnetic resonance phenomenon. After the RF excitation dissipates, the deviated atomic nucleus slowly returns to the initial state due to the action of the static magnetic field, inducing an induced electric signal in the coil, that is, the NMR signal. This signal is amplified by the low-noise amplifier [41] after passing through the duplexer to facilitate subsequent circuit processing. The spectrum analyzer circuit converts the data and transmits it to the computer for data processing.

**Figure 8:**
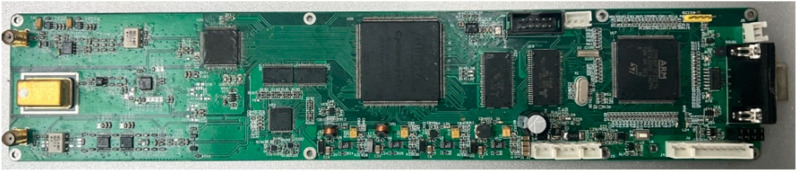
The real spectrometer.

The function of the microprocessor is to facilitate communication between the upper and the lower computer, coordinate the upper computer protocol and communication with the FPGA, and process information. The main function of the FPGA is to control the pulse transmission circuit and signal reception process, demodulate and filter the signal output from the ADC, and store the data. The pulse transmission circuit directs the DDS chip through the FPGA to generate a sequence of waveforms with different parameters such as frequency, amplitude, phase, echo time, and pulse duration. After the echo signal is amplified by a low-noise amplifier and VGA (Variable-gain Amplifier) circuit, it is received by the ADC, transmitted to the FPGA, and then internally extracted by the signal demodulation processing module. The real spectrometer is shown in Figure 8.

**Figure 9:**
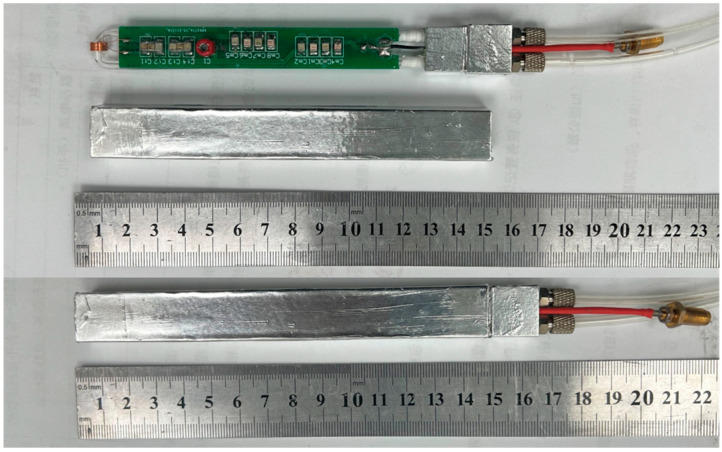
The real NMR probe.

The probe in the NMR magnetometer is responsible for transmitting and receiving radio frequency (RF) pulses and NMR signals. The NMR probe uses a π-type matching circuit that can adjust the position of the resonance frequency point through the adjustable capacitor. When the NMR signal is transmitted from the receiving coil to the preamplifier, from the perspective of energy utilization, the NMR signal can ideally be transmitted to the preamplifier as completely as possible. Therefore, the output impedance of the coil should be matched with the input impedance of the preamplifier. Otherwise, the NMR signal reflection will occur at the transmission port, making the already weak low-strength field NMR signal even weaker. The real spectrometer is shown in Figure 9.

## 4. Experiment

**Figure 10:**
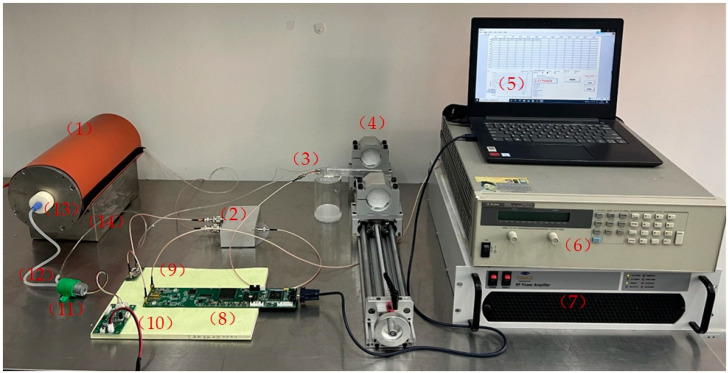
NMR magnetometer and permanent magnet measurement platform.

As described in the above introduction, we built a flow-style NMR magnetometer to measure two types of test platforms and analyze the results. The physical diagram of the 8mT measurement platform with permanent is shown in Figure 10.

The 30 mT permanent magnetic field measurement platform includes: (1) a pre-polarization magnet, (2) a duplexer, (3) an NMR magnetometer probe, (4) an r permanent, (5) a PC, (6) a high-precision current source for the solenoid coil to be measured (not used in permanent magnet measurements), (7) an RF power amplifier, (8) a spectrometer, (9) a low-noise preamplifier, (10) a water pump control circuit, (11) a water pump, (12) a pipe, (13) a water tank, and (14) a magnet base.

**Figure 11:**
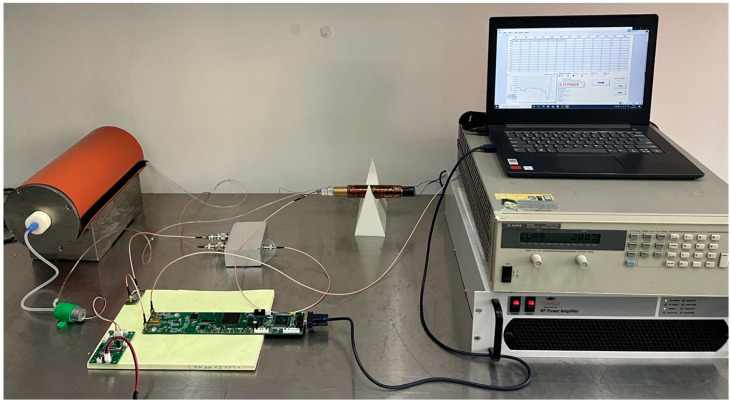
NMR magnetometer and solenoid coil measurement platform.

As shown in Figure 11, the magnetic field measurement platform for the solenoid coil is essentially the same as the platform for the permanent, with the main difference being that the area to be measured is replaced by a solenoid coil that has good magnetic field homogeneity. By adjusting the distance between the two permanent magnets, the magnetic field can be adjusted from 8 mT to 200 mT, but the magnetic field homogeneity remains low. The solenoid coil has good time stability, but the range of magnetic field adjustment is narrow. The magnetic field range can be adjusted from 0 to 10 mT by changing the output of the current source.

**Figure 12:**
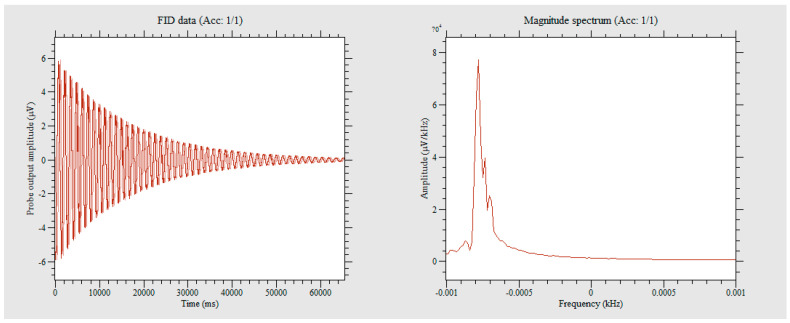
Measurement results of a 30mT magnetic field generated by the permanent magnet.

As shown in Figure 12, by measuring the magnetic field of a permanent magnet with a strength of 30 mT, the FID signal was determined for specific field strength. The signal was then Fourier transformed to obtain the frequency spectrum peak, which was found to be accurate to within 0.5 Hz. The 0.5 Hz corresponds to a magnetic field strength of 11 nT, indicating that the measurement accuracy of this method at 30 mT is much better than 11 nT.

**Figure 13:**
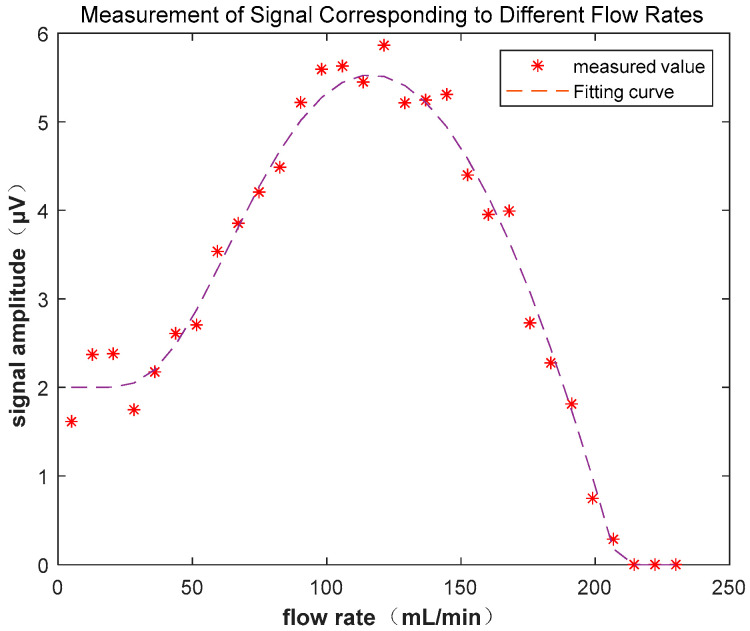
Measurement of signal corresponding to different flow rates.

In the 30 mT platform, signals of different flow rates were acquired, as shown in Figure 13, which illustrates the changes in the peak of the FID signal as the flow rate increased. Initially, due to the low flow rate, the polarized water sample had not yet flowed into the detection area and had already depolarized, so the measured magnetic resonance signal was the FID signal of the depolarized water sample for the 30 mT magnetic field. The detected signal was relatively weak. As the flow rate increased, the pre-polarized water sample flowed into the detection area, increasing the strength of the FID signal, and the optimal flow rate to maximize the signal could be determined. As the flow rate continued to increase, the portion of the water sample excited in the detection coil was not detected before being flushed out of the detection area, and no voltage signal was induced, resulting in a signal amplitude of zero. By fitting the measured waveform of flow rate and signal amplitude, the ratio of the signal amplitude at the optimal flow rate to the signal amplitude at rest was approximately 2.69, which is consistent with the results of the simulation analysis. Moreover, the image trend matched the physical simulation.

**Figure 14:**
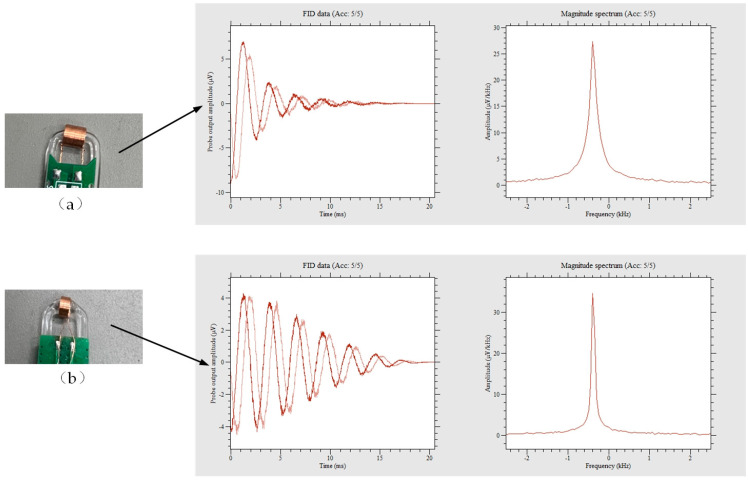
Measurement results for different probe coils (Picture (**a**) shows that the probe coil has a diameter of 5 mm and a length of 7 mm. Picture (**b**) shows that the probe coil has a diameter of 5mm and a length of 3 mm).

In addition, to investigate the measurement capability of different probes in inhomogeneous fields, a comparative experiment was conducted on probes with different coil lengths. Except for the difference in coil length, all other equipment was identical, and the measured magnetic field and flow rate were the same. As shown in Figure 14, through the experiment, it was found that although longer coil lengths increased the signal peak of the FID signal in the time domain, the longer the probe coil length, the faster the FID signal decayed, resulting in a slower FFT spectrum peak and a smaller peak-to-width ratio. On the contrary, the smaller the probe, the slower the FID signal decayed and the sharper the spectrum. The reason is that the longer the probe coil, the wider range of magnetic fields it involves in the measured magnetic field, and the measured magnetic field is inevitably inhomogeneous. An inhomogeneous magnetic field induces signals of different frequencies in the probe, significantly reducing *T*_2_*, resulting in faster signal decay. This proves that the homogeneity of the magnetic field is crucial to the NMR signal, which can inform the design of meter probes, as smaller probes can provide better measurement capability for uneven magnetic fields while also improving the spatial resolution of the measured magnetic field.

**Figure 15:**
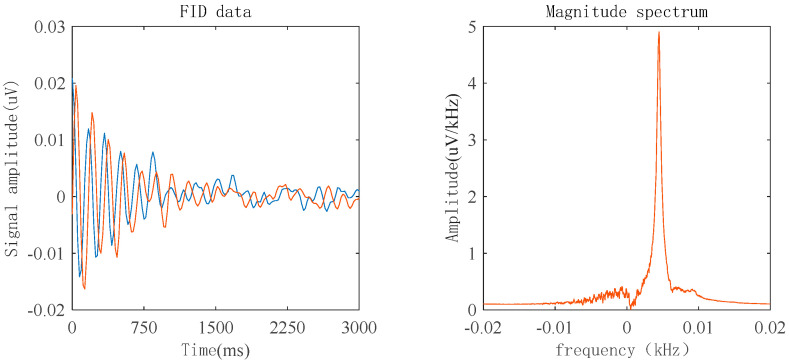
Measurement results of an 8 mT magnetic field generated by the solenoid coil. (in the left image, The blue line represents the imaginary part data, and the yellow line represents the real part data).

As shown in Figure 15, for the 8 mT solenoid coil measurement platform, the magnetic field strength needs to be reduced for measurement, which leads to a decrease in the signal-to-noise ratio of the FID signal. In performing FFT transformation on the signal, it was found that the peak curve became relatively flat. The peak values were then extracted using a computer program, and the average peak values were found to be within a 1 Hz range; therefore, its accuracy is around 1 Hz.

## 5. Conclusions

In this work, we provide a detailed explanation of the measurement principles of a pulse-wave DNP NMR magnetometer. We analyze the measurement process and signals output and discuss the influence of various system parameters on the measurement results by constructing a mathematical model for pre-polarization detection of water samples. We complete the system design of an entire flow-type NMR magnetometer and validate it experimentally on two magnetic field test platforms. The results show that pre-polarization processing increased the NMR signal strength by 2.69 times, which verifies the relationship between signal amplitude and flow velocity and represents a measurement accuracy within 1 Hz at 8 mT and 0.5 Hz at 30 mT. However, there are still some issues with the device, such as the narrow bandwidth of the probe, which requires customization of the probe according to different magnetic field ranges. Follow-up work will continue to optimize the probe structure and improve the device’s wide-range measurement capability.

## Figures and Tables

**Table 1:** Comparison between previous work and our work.

Literature Work	Experimental Magnetic Field Strength	Measurement Accuracy	Sample	Measurement Time	Spatial Resolution
Pendlebury (1979)	250 mT	unknown	water	unknown	unknown
Woo (1997)	46.985 mT	320 nT	water	>4 min	>1.2 cm^3^
Davydov (2015)	10.9 mT	0.04 mT	a distillate water isolated with methanol	4 min	>1 cm^3^
Michal Ulvr (2018)	2.3 mT	57.5 nT	water	30 min	<0.5 cm^3^
This paper	8 mT	22 nT	pure water	<10 s	<0.5 cm^3^

## Data Availability

Data underlying the results presented in this paper are not publicly available at this time but may be obtained from the authors upon reasonable request.
